# Mucosal-Associated Invariant T Cells in Multiple Sclerosis: The Jury is Still Out

**DOI:** 10.3389/fimmu.2015.00503

**Published:** 2015-09-30

**Authors:** Emmanuel Treiner, Roland S. Liblau

**Affiliations:** ^1^Centre de Physiopathologie de Toulouse-Purpan (CPTP), INSERM UMR1043-CNRS 5282, Toulouse, France; ^2^Université Toulouse III - Paul-Sabatier, Toulouse, France; ^3^Department of Immunology, Toulouse University Hospital, Toulouse, France

**Keywords:** multiple sclerosis, adaptive immunity, innate-like T cells, MHC-related 1, IFN gamma

## Abstract

The immune system is strongly implicated in the pathophysiology of multiple sclerosis (MS), as demonstrated by the efficacy of therapies targeting various components of adaptive immunity. However, the disease still progresses despite these treatments in many patients, while others experience life-threatening adverse effects, urging for the discovery of new immune-targeting medications. Among the immune cell types participating to MS pathogenesis, decades of work have highlighted the prominent role of CD4 T cells. More recent data demonstrate the involvement of CD8 T cells as well. The existence of both pathogenic and protective CD8 T cells subsets has been suggested, adding an additional layer of complexity to the picture. Mucosal-associated invariant T (MAIT) cells are innate-like lymphocytes that make up to 25% of CD8 T cells in healthy subjects. They are specific for conserved microbial ligands and may constitute an important barrier against invasive bacterial and fungal infection. An increasing number of reports also suggest their possible involvement in chronic inflammatory diseases, including MS. MAIT cells could participate through their ability to produce IFNγ and/or IL-17, two major cytokines in the pathogenesis of several chronic inflammatory/autoimmune diseases. However, the mechanisms by which MAIT cells could be activated in these sterile conditions are not known. Furthermore, contradictory observations have been made, reporting either a protective or a pro-inflammatory behavior of MAIT cells in MS or its murine model, experimental autoimmune encephalomyelitis. In this review article, we will describe the current knowledge on MAIT cell biology in health and disease, and discuss the possible mechanisms behind their role in MS. The specific features of this new non-conventional T cell subset make it an interesting candidate as a biomarker or as the target of immune-mediated intervention.

Multiple sclerosis (MS) is a chronic immune-mediated disease of the central nervous system (CNS). MS is characterized by discrete white matter lesions in the brain and spinal cord. The cellular injury is primarily oligodendrocytes but MS also affects axons/neurons. This neural demise results in the progressive neurological disability affecting people with MS. The genetic factors providing susceptibility to the disease have been largely deciphered in recent years (1). Polymorphisms at more than 150 loci contribute to MS susceptibility. Importantly, the incriminated genes collectively point to a central role of the immune system in disease pathogenesis (2, 3). The most studied and also the strongest MS-susceptibility genes reside within the human leukocyte antigen (HLA) locus with a major influence of the DRB1*15:01-HLA-DRB5*01:01-DQB1*06:02 haplotype and significant impact of HLA class I alleles (4).

There is little doubt that multiple immune cell populations are implicated both at the initiation of the disease process and at the effector phase responsible for CNS tissue damage. However, the respective contribution of these various populations at the different phases of the disease remains only partly understood (5). Nevertheless, deciphering how the various CD4 and CD8 T cell subsets promote and regulate MS immunopathogenesis has benefited from progress in fundamental immunology and from experimental models (6–8). Much has been learned lately regarding the different functional subsets of CD4 T cells and regarding the pathogenic and regulatory influence of CD8 T cells. This has, in part, led to new therapeutic directions for the benefit of people with MS (9). However, newly identified innate-like T cell populations, such as innate lymphoid cells, invariant natural killer T (iNKT) cells, and mucosal-associated invariant T (MAIT) cells, have emerged as important actors in inflammatory diseases. They are positioned at the interface between the environment and the host and may, therefore, represent a key link for the amplification of an immune reaction against microbes. Understanding their exact contribution to pathogenesis will undoubtedly open innovative therapeutic possibilities.

Here, we review the current knowledge regarding the biology of MAIT cells and their possible involvement in MS. Future directions are suggested to better apprehend their precise role and their usefulness as therapeutic targets.

## Mucosal-Associated Invariant T Cells: A New Innate-Like T Cell Subset

**Figure 1:**
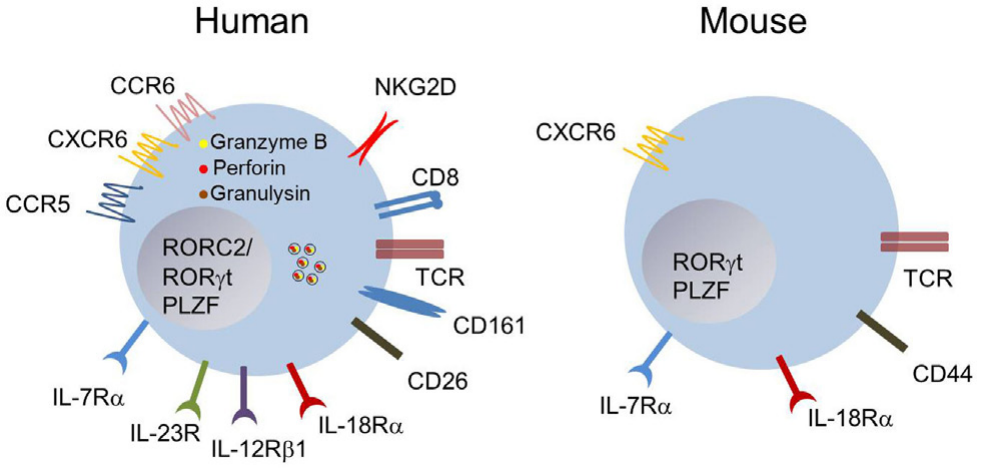
**Phenotype of human and mouse MAIT cells**. Blood MAIT cells in humans are defined as TCRVα7.2^+^CD161^hi^-expressing T cells. Most MAIT cells are CD8^+^, and express an effector/memory phenotype CD45RO^+^CD62L^lo^CCR7^−^ (not depicted). They express several chemokine- and interleukin-receptors at steady state. They are equipped with the cytotoxicity co-receptor NKG2D, and display intra-cytoplasmic granules containing granulysin, granzyme B, and perforin. The expression of CD161, IL-18Rα, or CD26 at high levels is usually sufficient to identify MAIT cells within the CD8^+^ subset in human blood. The phenotype of mouse MAIT cells is apparently more diverse, and dependent upon the tissues examined. Most of them are CD4^−^CD8^−^ (double negative), display an effector/memory phenotype and the interleukin and chemokine receptors IL-7Rα, IL-18Rα, and CXCR6 (16, 17, 23, 24, 32).

**Figure 2:**
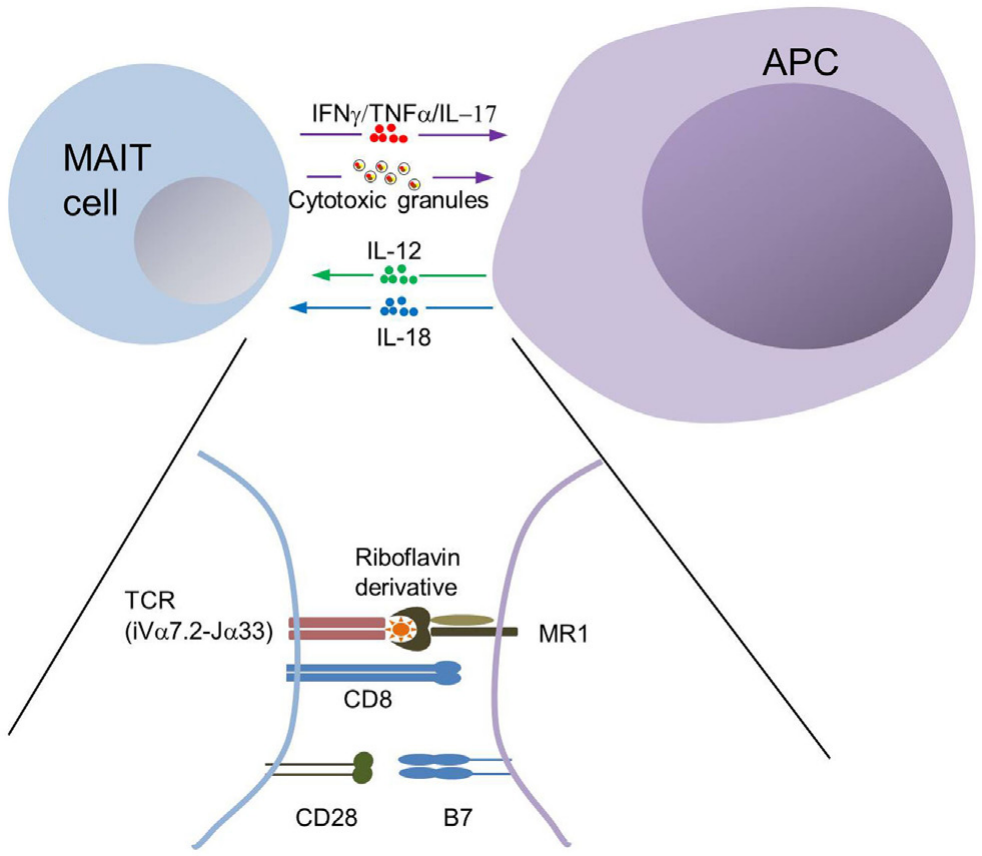
**MAIT cell interactions with MR1^+^ antigen-presenting cells**. The semi-invariant TCR of MAIT cells allows recognition of a complex made of MR1 associated with a bacterial-derived riboflavin derivative. MR1-dependent interactions with MAIT cells has been formerly demonstrated with professional antigen-presenting cells (APCs), such as monocytes, macrophages, dendritic cells, and B cells as well as with epithelial cells. Professional APCs may influence MAIT cell response through secretion of IL-12 and IL-18, and by providing co-stimulation. MAIT cell effector functions primarily involve IFNγ secretion and degranulation of their cytotoxic content, thereby inducing killing of infected epithelial cells.

Mucosal-associated invariant T cells are a homogenous T cell subset displaying features of innate-like T cells, such as γδ or iNKT cells. Originally described in humans, they are phylogenetically conserved in distant mammal species, including mice (10–14). However, the frequency of MAIT cells in laboratory mouse strains is low, and there is evidence that they may be developmentally and/or functionally different from their human counterparts (15). These differences must be kept in mind when interpreting results obtained in mice. MAIT cells are mainly characterized by a highly restricted TCR repertoire, selected for by a monomorphic major histocompatibility complex (MHC) class I-like molecule known as MHC-related 1 (MR1) (10). Indeed, the vast majority of MAIT cells express an invariant TCRα chain (Vα7.2-Jα33 in humans and the homologous Vα19-Jα33 in mice) (13, 16). The second important feature of MAIT cells is their peripheral maturation/differentiation status; in one study, >90% of MAIT cells displayed an effector/memory phenotype in healthy adults (17). The ontogeny of MAIT cells in mice is dependent upon microbial colonization of the intestine soon after birth, suggesting that shared commensal bacterial antigens presented by MR1 drive the proliferation and maturation of memory MAIT cells (17). In humans, cord blood harbors a small population of naïve MAIT cells that apparently expand in early childhood, and differentiate into memory cells (17, 18), suggesting a similar mechanism of antigen-driven expansion after birth. Seminal studies performed by the Rossjohn and McCluskey laboratories led to the discovery of microbial antigens for MAIT cells (19, 20). These antigens are low molecular weight molecules derived from the intermediates of the riboflavin (vitamin B2) metabolism. The mammalian genome is devoid of the genes necessary to the synthesis of riboflavin; however, an important number of different bacterial and fungal species are riboflavin producers, and therefore, MAIT cell activators (21). It is, therefore, speculated that MAIT cells exit the thymus as naïve cells, and then encounter bacterial antigens (probably originating from the commensal flora), driving their early maturation in the periphery. However, a recent study challenged this hypothesis, showing evidences of MAIT cell proliferation and differentiation in the peripheral organs of second trimester human fetuses (22) This would suggest that MAIT cells can mature before bacterial colonization of the body with commensal microbes, and has profound consequences on our understanding of MAIT cells reactivity toward various cognate ligands and/or environmental cues; more studies are, therefore, needed to unravel these processes. Activation of MAIT cells leads to cytokine secretion, mostly interferon γ (IFNγ) and tumor necrosis factor α (TNFα), as well as induction of degranulation and cytotoxicity (23–25). Virtually all MAIT cells in humans express high levels of CD161, as well as the IL-23 receptor (IL-23R), the C-C chemokine receptor 6 (CCR6) and the transcription factor RAR-related orphan receptor gamma t (RORC2/RORγt), three markers associated with interleukin 17 (IL-17) producing subsets (Figure 1) (16, 18, 23). Indeed, IL-17-secreting MAIT cells can be found in some settings, mostly in pathological conditions (see below) (23, 26–31). The phenotype of MAIT cells in wild-type mice has been described recently (Figure 1). In peripheral tissues, such as the lung, they uniformly express a memory phenotype (CD44^hi^CD62L^lo^), the cytokine receptors IL-7R and IL18R, and the C-X-C chemokine Receptor 6 (CXCR6) chemokine receptor, akin to humans (32). Other surface markers show a more diverse pattern of expression, suggesting that mouse MAIT cells may be more diverse than their human counterparts. In particular, most mouse MAIT cells lack both CD4 and CD8, but variable proportions of CD4 and CD8 cells are found in a tissue-specific fashion (32). In agreement with their anti-microbial reactivity, MAIT cells react against a wide array of bacteria *in vitro*, by producing IFNγ and lysing bacteria-infected cells (19, 24, 25, 33–36). *In vitro* analysis showed that their functional response is regulated by several cytokines, such as IL-7, IL-12, IL-18, and IL-23 (Figure 2) (26, 30, 37, 38). MAIT cells may be particularly involved in the immune response against *Mycobacterium tuberculosis*: patients with active tuberculosis show a depleted blood MAIT cell compartment resulting from their recruitment to the lung (33, 38–40). All evidence points out an important role for MAIT cells as a first line of defense against invasive bacterial infections, chiefly at mucosal surfaces (41).

An increasing number of studies have reported the likely involvement of MAIT cells in non-bacterial diseases. These include chronic viral infections like human immunodeficiency virus (HIV) (42–48), autoimmune diseases [such as inflammatory bowel diseases (IBD), systemic lupus erythematosus (SLE), MS, or psoriasis] (27, 31, 49–52), other inflammatory or hypersensitivity diseases, and even cancer (28, 29, 53–56). Although the significance of these observations is still unknown, they point out a possible role for MAIT cells in the pathogenesis of many inflammatory conditions.

## MAIT Cells in MS and EAE

The first study focusing on MAIT cells in the context of CNS inflammation was performed in humans. Because at the time there was no tool available to directly identify MAIT cells (such as TCR-targeting antibodies or tetramers), the transcripts encoding the MAIT cell-specific invariant TCRα chain *V*α*7.2-J*α*33* were searched for within the CNS (49). An accumulation of such mRNA species was found in autopsy lesions from MS patients in 50% of the analyzed cases, as well as in 73% of cerebrospinal fluid samples obtained from patients experiencing relapses. The authors concluded that MAIT cells are involved in the CNS inflammation. The same group further investigated the role of MAIT cells in the EAE mouse model. As MAIT cells are scarce in mice, they increased their number and frequency by generating a mouse transgenic (Tg) for the MAIT cell-specific TCRα chain (iVα19). Upon immunization with a myelin oligodendrocyte glycoprotein (MOG) peptide (57), iVα19 Tg mice showed dramatically reduced EAE incidence and severity, as compared with wild-type B6 or CD1d-deficient mice, which lack CD1d-restricted iNKT cells. A similar regulatory effect of MAIT cells was observed in regular B6 mice adoptively transferred with iVα19 Tg cells, as well as in MR1 knock-out (KO) mice compared to wild-type animals. iVα19 Tg cells induced IL-10 production by B cells in a MR1-independent manner *in vitro*, accompanied by a reduction in the production of inflammatory cytokines by MOG-specific T cells, probably accounting for the EAE-protecting effect of MAIT cells. Altogether, these data strongly suggested that MAIT cells display an immune-regulatory function in the context of EAE. The interpretation of these data was, however, hampered by the fact that iVα19 Tg mice were not crossed onto a Cα KO background, thereby allowing endogenous TCRα chains to be recombined and expressed. It is, therefore, difficult to evaluate how the alterations induced in the TCR repertoire of iVα19 Tg mice could have impacted the data obtained in the EAE model.

However, this striking paper triggered several studies evaluating MAIT cell numbers and functions in MS patients, which yielded contradictory results. Miyazaki et al. observed a dramatic reduction in the frequency of blood MAIT cells in patients with relapsing-remitting MS (RR-MS) (58). The frequency of blood MAIT cells inversely correlated with disease activity as it was lower in active disease as compared to stable patients. Interestingly, steroid treatment of active patients induced a rise in the frequency of MAIT cells. This may suggest that a reversible, altered distribution could be responsible for their depletion from blood in the acute phases of inflammation. It was suggested from *in vitro* experiments that MAIT cells suppress IFNγ production by other T cell subsets, akin to the data obtained with iVα19 Tg mice; however, this suppression was independent of B cells or IL-10 production. By contrast, Annibali et al. observed an increased frequency of CD8^+^CD161^hi^ T cells in the blood of MS patients (51). Several independent studies have clearly demonstrated that this T cell subset is almost exclusively composed of MAIT cells (16, 18). CD8^+^CD161^hi^ T cells from MS patients displayed an inflammatory phenotype characterized by high IL-17 production, and could be found in post-mortem brain biopsies within perivascular cuffs and chronic active lesions. Thus, MAIT cells in these patients behaved oppositely from the previous reports, and suggested a pro-inflammatory pathogenic role in MS. These data were in part corroborated by an analysis of post-autologous hematopoietic stem cell transplantation in MS patients. This study showed that MAIT cells were depleted by a conditioning regimen including cyclophosphamide or alemtuzumab and did not recover for more than 2 years post-graft (59). Before immunosuppressive conditioning, the patients exhibited a high frequency of MAIT cells, together with a pro-inflammatory profile. Moreover, MAIT cells were identified within white matter inflammatory lesions from post-mortem samples of nine MS cases. In addition, a recent report described a significant but modest decrease of MAIT cells in the blood of RR-MS patients (60), and confirmed their presence in brain lesions. Stimulation of MAIT cells with IL-18 induced very late antigen 4 (VLA-4) up-regulation, providing a possible mechanism to explain their migratory behavior. In agreement with this, the decrease in blood MAIT cells was inversely correlated with IL-18 plasma levels (60). More recently, Held and collaborators made a strong effort to identify antigen-driven T cells expansion within brain lesions by combining laser microdissection and TCR pyrosequencing (61). They indeed observed massive expansion of T cell clones that persisted for several years. Surprisingly, TCRs closely related to, but distinct from, the MAIT cells’ TCR were found, whereas the canonical TCR Vα7.2-Jα33 was present as a minor fraction (61). It is not clear at this point to what extent these TCRs allow any recognition of MR1, and how T cells expressing these specificities are indeed related to MAIT cells. Thus, opposite results are described with regard to MAIT cell frequency and functions in MS patients, and the reasons underlying these discrepancies are currently not clear. It should be pointed out at this point that the effect of immunosuppressive/immunomodulatory regimen on MAIT cells has never been studied, with the exception of the hematopoietic stem cell transplantation study mentioned above. The cohorts analyzed in these various articles included patients that were free of any current medication affecting the immune system, but no mention of previous treatments was made, with the exception of the Annibali’s work, which analyzed mostly treatment-naïve patients. Therefore, differences in treatment history may hamper a valid comparison of these cohorts. Nevertheless, available data suggest that MAIT cells in MS gain the ability to traffic to the CNS, which in some cases may explain their depletion from blood, as seen in other pathological settings (see below).

## MAIT Cells in Other Autoimmune/Inflammatory Diseases

Several reports have described alterations in the frequency, phenotype, location and/or functions of MAIT cells in inflammatory diseases, mostly in human samples and sometimes in mouse models. Most studies found a decreased frequency of blood MAIT cells, in patients with SLE, celiac disease, or IBD (31, 52, 56). When studied, the function of MAIT cells was altered, with decreased IFNγ production in SLE and IBD, and increased IL-17 secretion in IBD. Increased MAIT cell frequency was found in the inflamed intestinal tissue of IBD patients as well as in skin lesions of patients with psoriasis (27, 31). MAIT cells appeared pathogenic in a mouse model of autoimmune arthritis (62). In this study, the authors showed that MR1 KO animals were less sensitive to both active (collagen-induced) and passive (antibody-induced) arthritis than wild-type animals. Moreover, they used adoptive transfer of MAIT cells from iVα19 Tg mice (with the limitations already described) to reveal the disease-enhancing role of MAIT cells in the passive arthritis model. Recently, two seminal studies documented the behavior of MAIT cells in obese patients. It appears that, similar to several autoimmune or infectious diseases, MAIT cell frequency gradually declines in the blood of obese patients, and increases in the adipose tissue where MAIT cells are prone to produce IL-17 (28, 29).

## Putative Mechanisms of MAIT Cells Involvement in MS and Other Inflammatory Diseases

The diverse and clonal expression of the TCR by T cells implies that different clones react against different ligands. MAIT cells display very limited repertoire diversity and therefore should recognize a limited set of different ligands. However, akin to other innate T cell subsets (such as γδ T cells or CD1d-restricted iNKT cells), the antigens recognized by MAIT cells are highly conserved and potentially expressed by a large variety of bacteria and fungi (34). This property accounts for the activation of MAIT cells by various microorganisms, such as *Escherichia coli*, *Mycobacterium tuberculosis*, *Klebsiella pneumoniae*, *Shigella flexnerii*, *Salmonella typhimurium*, *Vibrio cholerae*, or *Francisella tularensis*, and certainly many others (20, 24, 33–35, 63–66).

However, our knowledge of the mechanisms by which MAIT cells are recruited and activated in non-infectious inflammatory diseases is minimal. The first general hypothesis postulates that MAIT cells are activated in a cognate manner, by recognition of their specific Ags. But what would be the origin of these antigens? In the case of intestinal inflammatory diseases, alterations in the permeability of the gut epithelial barrier and subsequent translocation of microbial products may promote MAIT cell activation. Further, recent findings suggest that this general mechanism is involved in the pathogenesis of non-intestinal inflammatory diseases, including MS (67, 68). However, it would be difficult to explain how a low level of microbial translocation would induce a strong MAIT cell activation, given the fact that their specific ligands appear to be extremely unstable (20), which could actually be a regulation mechanism. Alternatively, there may be endogenous ligands for MAIT cells, whose expression could be induced and/or increased in the context of inflammation. It must be pointed out that there is to date no demonstration that such endogenous ligands exist. However, this could be inferred from several studies analyzing MAIT cell ontogeny, which proved that MAIT cells are positively selected on hematopoietic cells in the thymus in the presence of MR1 only (10, 69). Thus, although it is highly probable that such endogenous ligands for MAIT cells exist, their molecular characterization is still eagerly awaited.

The second hypothesis to explain such broad involvement of MAIT cells in inflammatory diseases involves a bystander mechanism, i.e., in the absence of their antigen recognition through their TCR. MAIT cells are effector/memory cells, and as such, are equipped with a panel of receptors involved in cell migration, or in the response to inflammatory mediators [Toll-like receptors (TLR), cytokine receptors, etc.]. Notably, MAIT cells can be activated to produce IFNγ in the mere presence of cytokines, specifically a combination of IL-12 and IL-18 (37). Jo et al. showed that the TLR7/8 agonist R848 stimulates monocytes to produce IL-12 and IL-18, which in turn activate IFNγ secretion by MAIT cells (70). Further, bacteria devoid of MR1-binding ligands, such as *Streptococcus pneumoniae*, also induce the production of these cytokines by antigen-presenting cell (APC) and subsequent MAIT cell activation. IL-12 is involved in many autoimmune diseases, including MS (and EAE); IL-18 is also a major cytokine whose role in driving autoimmune diseases but also hypersensitivity conditions is gaining strong interest (71, 72). Therefore, it could be proposed that the inflammatory milieu drives MAIT cell activation through cytokine responsiveness, leading to their recruitment in the inflamed lesions of the brain in MS. Indeed, IL-18 apparently up-regulates VLA-4 at the surface of MAIT cells, providing a molecular clue as to how the cytokine milieu might influence MAIT cells migratory behavior (60). Other cytokines, such as IL-1β, which shares with IL-18 an inflammasome-dependent processing, might also be involved.

## Possible Roles of MAIT Cells in the Inflamed Brain during MS

Mucosal-associated invariant T cells can be found in the inflamed CNS lesions from MS patients; they are also observed in other inflamed tissues, such as the skin of psoriatic patients. This raises the obvious question of their functions within tissues and their relevance to the pathogenesis of these diseases in general. Very little information is available about MAIT cell functions in tissues, and we are currently led to speculate on this matter. It is of course possible that they are only innocent bystanders in the inflammatory lesions and do not play any important role. Blood MAIT cells are equipped with receptors involved in migration to the CNS, such as CCR5 (in the steady-state) and VLA-4 (after stimulation). Indeed, MAIT cells are usually identified in low frequency within target tissues of inflammation. On the other hand, it must be reminded that MAIT cells are effector/memory cells prone to produce inflammatory cytokines and to release cytotoxic granules. Several reports showed that circulating blood MAIT cells in diseases, such as IBD, type 2 diabetes (T2D), and MS, display increased cytokine-producing functions, in particular IL-17, as compared with their counterparts from healthy donors (29, 31, 51). Therefore, infiltrating MAIT cells in MS are functionally active, which may suggest their involvement in the disease. If so, the next question is: what role do MAIT cells play in the inflamed brain?

Upon activation, cytokine secretion by MAIT cells is mostly related to a Tc1/Tc17 pattern, i.e., IFNγ and/or IL-17 as well as GM-CSF and TNFα. All these cytokines are considered as major culprits in many autoimmune diseases, including MS. From there, it is tempting to speculate that MAIT cells are pro-inflammatory cells with deleterious effects in the disease process. Furthermore, given that MAIT cells are cytotoxic against bacteria-infected epithelial cells, it is possible that in MS they gain the ability to kill oligodendrocytes, axons/neurons, or even other CNS-resident cell types. Direct evidence supporting this hypothesis is, however, lacking. Although MR1 (the restriction molecule for MAIT cells) appears to be broadly expressed, its expression in CNS cells has never been investigated. This aspect needs to be addressed in MS as well as in other CNS inflammatory diseases. We think that the current knowledge regarding the functional properties of MAIT cells does not permit to predict whether these cells ultimately play a pro-inflammatory or regulatory role in MS. MS is probably a heterogeneous disease with regard to the precise mechanisms of pathogenesis. Although the issue is not totally resolved, there is increasing evidence that both inflammasome-dependent and inflammasome-independent mechanisms exist that would be differentially elicited in patients, and that this may underlie the differential response to therapy (73, 74). We postulate that this heterogeneity in the disease pathogenesis could partially explain the contradictory results found by different teams with regard to the pro-inflammatory or regulatory role of MAIT cells in MS and EAE. If this hypothesis holds true, we also anticipate that there could be a correlation between MAIT cells functions in MS (for instance, IFNγ versus IL-17 production) and response to disease-modifying therapies, suggesting a possible use as an immunological biomarker.

## Concluding Remarks

The multifaceted nature of MAIT cells makes them promising candidates for therapeutic targeting and/or to use as biomarker of disease. A strong body of work strongly suggests that MAIT cells are involved in MS, fostering new studies aiming at deciphering their precise role in pathogenesis. Studies in human patients are obviously hampered by the limited access to tissue samples. One interesting avenue would be to analyze MAIT cells phenotype and functions in patients stratified according to their response to therapy. Limitations of the murine models available thus far have been already described. However, a report published while this review was in preparation described MAIT cells in wild-type mice, with the help of MR1 tetramers (32). The authors suggest that mouse MAIT cells more closely resemble their human counterparts than previously thought, although important differences remain, such as a much lower frequency and a more pronounced Tc17 skewing of cytokine secretion. There is no doubt that future analysis of MAIT cells in the EAE model will yield relevant data as to their role in this disease.

## Conflict of Interest Statement

The authors declare that the research was conducted in the absence of any commercial or financial relationships that could be construed as a potential conflict of interest.
